# Relations Between Behavioral Inhibition, Big Five Personality Factors, and Anxiety Disorder Symptoms in Non-Clinical and Clinically Anxious Children

**DOI:** 10.1007/s10578-012-0302-5

**Published:** 2012-04-17

**Authors:** Leonie J. Vreeke, Peter Muris

**Affiliations:** 1 Institute of Psychology, Erasmus University Rotterdam, Burgemeester Oudlaan 50, Suite T12-35, PO Box 1738, 3000 DR Rotterdam, The Netherlands; 2 Department of Clinical Psychological Science, Maastricht University, Maastricht, The Netherlands

**Keywords:** Behavioral inhibition, Anxiety disorder symptoms, Neuroticism, Extraversion

## Abstract

This study examined the relations between behavioral inhibition, Big Five personality traits, and anxiety disorder symptoms in non-clinical children (*n* = 147) and clinically anxious children (*n* = 45) aged 6–13 years. Parents completed the Behavioral Inhibition Questionnaire-Short Form, the Big Five Questionnaire for Children, and the Screen for Child Anxiety Related Emotional Disorders-Revised. Results indicated that, compared to parents of non-clinical children, parents of clinically anxious children rated their offspring higher on neuroticism and behavioral inhibition, but lower on extraversion, conscientiousness, and intellect/openness. Further, extraversion emerged as the strongest correlate of an inhibited temperament, and this appeared true for the clinically anxious as well as the non-clinical children. Finally, in both the clinical and non-clinical samples, higher levels of behavioral inhibition and neuroticism were unique and significant predictors of anxiety disorders symptoms.

## Introduction

Some people run greater risk for developing anxiety disorders than others. Two personality traits that seem to be particularly relevant for understanding this enhanced vulnerability for anxiety problems are neuroticism and extraversion. Individuals scoring high on neuroticism are emotionally instable and as such would display a proneness to experience negative emotions such as fear and anxiety. Not surprisingly, neuroticism has been regarded as a predisposition to develop all kinds of psychopathology, including the anxiety disorders [1]. The personality trait of extraversion is concerned with the tendency of being concerned with or obtaining gratification from the external environment. Individuals high on this trait are generally sociable, enthusiastic, lively, and assertive. There is evidence indicating that low extraversion (i.e., introversion) is closely associated with anxiety problems [2, 3], which is not surprising as it is easy to see how this personality feature promotes avoidance behavior. Thus, a personality characterized by a combination of high levels of neuroticism and low levels of extraversion could be seen as a vulnerability factor for developing anxiety disorders in adults [4] and youths [5, 6].

It is of interest to note that this constellation of personality factors can already be identified at a fairly young age. More precisely, Kagan [7] described a temperament typology in toddlers, which referred to the tendency to react with shyness, fear, and withdrawal in response to novel or challenging situations. There is ample evidence that this typology of behavioral inhibition should be viewed as a vulnerability factor, which puts children at risk for developing anxiety disorders [8].

Previous research in 8- to 13-year-old non-clinical children [9, 10], examining the underlying personality factors of behavioral inhibition, has indeed confirmed the notion that behavioral inhibition is best characterized by high levels of neuroticism and low levels of extraversion. Further, it was found that even after controlling for the influence of neuroticism, extraversion and other Big Five personality factors, behavioral inhibition was still positively associated with anxiety symptoms, which underlines the importance of this temperamental variable in the pathogenesis of childhood anxiety problems. However, these earlier studies solely relied on samples of non-clinical children, and so it remains to be seen whether this pattern of results also emerges in clinically anxious children. Replication of these findings in a clinical population seems important as this would further underline the unique role of behavioral inhibition beyond basic personality traits in childhood anxiety disorders. With this in mind, the current study was conducted. Parents of non-clinical (*n* = 147) and clinically anxious (*n* = 45) children completed questionnaires measuring behavioral inhibition, Big Five personality traits, and anxiety disorder symptoms in their offspring. In this way, it became possible to investigate differences between non-clinical and clinically anxious children with regard to (a) levels of behavioral inhibition and Big Five personality traits, and (b) the pattern of correlations among behavioral inhibition, personality traits, and anxiety disorder symptoms. Further, within the samples of non-clinical and clinically anxious children it was examined (c) to what extent neuroticism, extraversion, and other personality traits accounted for unique variance in behavioral inhibition, and (d) whether behavioral inhibition explained unique variance in anxiety disorder symptoms beyond Big Five personality traits.

## Method

### Participants and Procedure

Parents of 380 children from four primary schools in the neighborhood of Rotterdam, The Netherlands, were approached by mail. In the letter, parents received information about the study and were asked to provide consent for their participation in the study. Parents who agreed to participate filled out the set of questionnaires and returned them to the researchers in a reply-paid envelope. One-hundred-and-fifty-five parents (41 %) responded positively to the mailing. Due to missing variables, questionnaires of 147 parents (125 mothers, 17 fathers, 1 both parents, and 4 other caretakers) were eventually used for the data analyses. The children in this non-clinical sample (74 boys and 73 girls) had a mean age of 9.07 years (SD = 1.65, range 6–13). Parents reported no clear signs of anxiety or other psychopathology for these children, although this was not formally checked by means of a standardized interview. As these children were not referred to a clinic for psychological help, they were defined as ‘non-clinical’.

Additionally, 45 parents of children who were referred to a specialized academic anxiety clinic filled out the questionnaires (in all cases this concerned the mother as she was always present with the child during the visit at the clinic). These children (18 boys and 27 girls) had a mean age of 9.47 years (SD = 1.42, range 6–12). Clinical diagnosis was determined by interviewing parents with the Dutch translation of the *Anxiety Disorders Interview Schedule for Children* (ADIS-C)*, parent version* [11, 12]. The ADIS-C is a semi-structured interview designed specifically for diagnosing anxiety and other common disorders in children and adolescents. The children in this sample had a primary diagnosis of separation anxiety disorder (*n* = 4), social anxiety disorder (*n* = 11), specific phobia (*n* = 12), generalized anxiety disorder (*n* = 5), and obsessive compulsive disorder (*n* = 1). Twelve children were diagnosed with an anxiety disorder not otherwise specified. Some children also met the criteria of other psychiatric disorders, such as depressive disorder (*n* = 5), dysthymic disorder (*n* = 2), oppositional defiant disorder (*n* = 2), and enuresis (*n* = 2).

### Questionnaires

The *Behavioral Inhibition Questionnaire* (BIQ) [13] is a 30-item parent-report instrument assessing Kagan’s [14] temperament characteristic of behavioral inhibition in three domains: social novelty (e.g., ‘My child is shy when first meeting new children’), physically challenging situations (‘My child is hesitant to explore new play equipment’), and situational novelty (e.g., ‘My child approaches new situations or activities very hesitantly’). Parents rate all items on a 6-point Likert scale, ranging from 1 (hardly ever) to 6 (almost always). In the present study, the 14-item short form of the BIQ, (i.e., the BIQ-SF) was used [15]. The BIQ and the BIQ-SF have been shown to possess good reliability and validity [13, 15], and this is also true for the Dutch versions of these scales [16].

The *Big Five Questionnaire for Children* (BFQ-C) [17] is a 65-item scale assessing the five basic traits of personality (i.e., the Big Five) in children and adolescents: (1) extraversion, which refers to energy, positive emotions and the tendency to seek stimulation in the company of others, enthusiasm, assertiveness, and self-confidence (e.g., ‘My child easily makes friends’), (2) agreeableness, which pertains to an inclination to be compassionate and cooperative towards others (e.g. ‘My child understands when others need my help’), (3) conscientiousness, which has to do with self-discipline, orderliness, precision, and the fulfillments of commitments (e.g., ‘My child likes to keep all my school things in a great order’), (4) neuroticism, which reflects the tendency to easily experience unpleasant emotions such as anxiety, depression, discontent, and anger (e.g., ‘My child easily gets angry’), and (5) intellect/openness, which has to do with intellectual skills and an appreciation for art, adventure, curiosity, and variety of experience (e.g., ‘‘My child knows many things’; ‘My child would like to travel and to know the habits of other countries’). Items are rated on a 5-point Likert scale, ranging from 1 (almost never) to 5 (almost always). Various studies found support for the psychometric properties of the BFQ-C [17–19].

The revised version of the *Screen for Child Anxiety Related Emotional Disorders*-*Revised* (SCARED-R) [20] was used. The SCARED-R is an extension of the original 41-item SCARED [21], which intends to measure symptoms of anxiety disorders in youths. Muris, Merckelbach, Schmidt, and Mayer [22] adapted the original SCARED by adding items in an attempt to measure symptoms of the entire spectrum of DSM-defined anxiety disorders in children and adolescents. Thus, the resulting SCARED-R consists of 69 items assessing symptoms of panic disorder, separation anxiety disorder, generalized anxiety disorder, social phobia, specific phobias, obsessive–compulsive disorder, and traumatic stress disorder. Parents have to indicate how frequently their child experiences each anxiety symptom on a 3-point Likert scale, with almost never = 0, sometimes = 1, and often = 2. Research has indicated that the SCARED-R is a reliable and valid questionnaire for measuring childhood anxiety problems [20, 22, 23].

### Data Analysis

Data were analyzed by means of the Statistical Package of Social Sciences (SPSS). Cronbach’s alphas were computed to examine the reliability of the questionnaires. *T* tests were conducted to explore sex differences and to compare non-clinical and clinically anxious children on various measures. Further, partial correlations (controlling for the influence of sex) were calculated among behavioral inhibition, anxiety symptoms, and personality traits in non-clinical as well as clinically anxious children. Finally, hierarchical regression analyses were carried out to examine (a) the unique contributions of the various personality traits to behavioral inhibition, and (b) the contribution of behavioral inhibition and various personality traits to anxiety symptoms in both non-clinical and clinically anxious children.

## Results

### General Findings

All questionnaires proved to be reliable in terms of internal consistency. That is, Cronbach’s alphas for the various questionnaires were all between .75 and .92 (see Table 1). Further, significant sex differences were observed for a number of Big Five personality traits. More specifically, in the non-clinical sample, parents rated girls as more conscientious [*M* = 34.79, SD = 6.02 for girls vs. *M* = 32.25, SD = 6.30 for boys; *t*(145) = 2.50, *p* < .05], whereas they reported boys as more neurotic [*M* = 22.08 SD = 6.40 for boys vs. *M* = 19.80, SD = 5.52 for girls; *t*(145) = 2.31, *p* < .05]. In the clinically anxious sample, girls were rated as more agreeable [*M* = 37.74, SD = 4.22 for girls vs. *M* = 34.13, SD = 5.14 for boys; *t*(43) = 2.49, *p* < .05] but lower on intellect/openness [*M* = 30.04, SD = 5.57 for girls vs. *M* = 33.41 *SD* = 5.46 for boys; *t*(43) = 2.00, *p* < .05]. No significant differences between boys and girls were found for behavioral inhibition and anxiety disorder symptoms.

**Table 1:** Means (standard deviations) and Cronbach’s alphas for various questionnaires, as completed by parents of non-clinical and clinically anxious children

	Non-clinical children (*n* = 147)		Clinically anxious children (*n* = 45)	
	*M* (SD)	α	*M* (SD)	α
BIQ	32.83 (11.61)a	.92	49.29 (14.16)b	.90
BFQ-C extraversion	36.37 (5.54)a	.79	30.56 (5.38)b	.75
BFQ-C agreeableness	37.31 (5.57)a	.87	36.30 (4.81)a	.81
BFQ-C conscientiousness	33.51 (6.27)a	.88	30.60 (6.34)b	.83
BFQ-C Neuroticism	20.95 (6.06)a	.86	23.74 (6.48)b	.87
BFQ-C intellect/openness	36.96 (6.76)a	.86	31.39 (5.72)b	.77
SCARED-R	20.50 (13.68)a	.92	37.89 (15.57)b	.91

*BIQ* Behavioral Inhibition Questionnaire, *BFQ*-*C* Big Five Questionnaire for Children, *SCARED*-*R* Screen for Child Anxiety Related Emotional Disorders-Revised. Means with different subscripts differ at *p* < .05

### Differences Between Non-Clinical and Clinically Anxious Children

As can be seen in Table 1, significant differences were found between non-clinical and clinically anxious children on various parent-rated questionnaires. As expected, parents rated clinically anxious children as significantly higher on behavioral inhibition [*t*(190) = 7.86, *p* < .001) and anxiety (*t*(190) = 7.22, *p* < .001]. In addition, parents rated clinically anxious children as higher on neuroticism [*t*(190) = 2.65, *p* < .05], but lower on extraversion [*t*(190) = 6.20, *p* < .001], conscientiousness [*t*(190) = 2.72, *p* < .05], and intellect/openness [*t*(190) = 5.00, *p* < .001] than their non-clinical counterparts.

### Correlations Among Behavioral Inhibition, Personality Factors, and Anxiety

Because of the observed sex differences for a number of variables, it was decided to perform a partial correlation analysis (which controlled for the influence of sex) to study the relations among the BIQ, BFQ-C, and SCARED-R. As can be seen in Table 2, a highly similar pattern was found for non-clinical and clinically anxious children. That is, behavioral inhibition was negatively correlated with extraversion (*r*s being −.50 and −.58 in the non-clinical and clinically anxious group, respectively) and agreeableness (*r*s being −.16 and −.40). Further, a substantial positive correlation was found between behavioral inhibition and anxiety disorder symptoms as indexed by the SCARED-R (*r*s being .47 and .52 in the non-clinical and clinically anxious group, respectively). In addition, in the non-clinical group, neuroticism was positively correlated with anxiety disorders (*r* = .39), while extraversion was negatively associated with such symptoms (*r* = −.24). In the clinically anxious group, comparable correlations were found (of respectively *r* = .23 and *r* = −.25), but here these relations were non-significant. The latter was probably due to the small sample size of this group, as tests for comparing correlation coefficients revealed no significant differences in the magnitudes of these correlations between non-clinical and clinically anxious children [both *t*(190) s ≤ 1.01, *p*s ≥ .31].

**Table 2:** Partial correlations (corrected for sex) between parent-rated questionnaires measuring behavioral inhibition, Big Five personality traits, and anxiety disorder symptoms, computed for non-clinical children (*n* = 147, below the diagonal), and clinically anxious children (*n* = 45, above the diagonal) separately

		1	2	3	4	5	6	7
1.	BIQ total score	–	−.58**	−.40*	.03	.01	−.08	.52**
2.	BFQ-C extraversion	−.50**	−	.45*	.27	.05	.43*	−.25
3.	BFQ-C agreeableness	−.16*	.49**	–	.59**	−.22	.29	−.21
4.	BFQ-C conscientiousness	.12	.30**	.55**	–	−.11	.47**	−.16
5.	BFQ-C neuroticism	.04	.02	−.35**	−.27**	–	−.17	.23
6.	BFQ-C intellect/openness	−.02	.41**	.40**	.61**	−.19	–	−.07
7.	SCARED-R total score	.47**	−.24*	−.17	−.05	.39**	−.13	–

*BIQ* Behavioral Inhibition Questionnaire, *BFQ*-*C* Big Five Questionnaire for Children, *SCARED*-*R* Screen for Child Anxiety Related Emotional Disorders-Revised

* *p* < .05

** *p* < .05/21

### Predicting Behavioral Inhibition from Personality Traits

Hierarchical regression analyses were carried out to examine the unique contributions of the various personality traits to behavioral inhibition as measured by the BIQ (while controlling for sex by entering this variable on step 1). In both the non-clinical and the clinically anxious group, extraversion was the strongest predictor of an inhibited temperament: as expected, beta values were negative (*β’*s being −.63 and −.54 in the non-clinical and clinically anxious group, respectively), indicating that lower levels of extraversion were associated with higher levels of behavioral inhibition (see Table 3). In addition, in the non-clinical group, neuroticism was found to make a small but unique positive contribution to behavioral inhibition (*β* = .16), which means that in these children, higher levels of neuroticism tended to be accompanied by higher levels of behavioral inhibition. This relation was not found in the clinically anxious group. The regression models further indicated that conscientiousness was positively related to behavioral inhibition in both groups (*β’*s being .31 and .36 in the non-clinical and clinically anxious group, respectively). Finally, in the clinically anxious group, agreeableness was negatively related to behavioral inhibition, which implies that higher levels of this personality trait are associated with lower levels of behavioral inhibition (*β* = −.42). Altogether, the personality traits accounted for 35 % of the variance in behavioral inhibition scores in the non-clinical group, and 48 % of the variance in this temperament characteristic in the clinically anxious group.

**Table 3:** Main results of the regression analyses predicting BIQ behavioral inhibition from Big Five personality traits

	B	SE	β	Δ*R*²
*Behavioral inhibition: non*-*clinical children* (*n* = 147)				
Step 1				.00
Sex	-.88	1.72	-.04	
Step 2				.35**
BFQ-C extraversion	−1.31	.18	−.63**	
BFQ-C agreeableness	.01	.20	.01	
BFQ-C conscientiousness	.57	.18	.31*	
BFQ-C neuroticism	.30	.15	.16*	
BFQ-C intellect/openness	.13	.16	.08	
*Behavioral inhibition: clinically anxious children* (*n* = 45)				
Step 1				.00
Sex	.70	4.24	.02	
Step 2				.48**
BFQ-C extraversion	−1.42	.39	−.54**	
BFQ-C agreeableness	−1.18	.50	−.42*	
BFQ-C conscientiousness	.81	.37	.36*	
BFQ-C neuroticism	.00	.29	.01	
BFQ-C intellect/openness	.25	.38	.10	

*BIQ* Behavioral Inhibition Questionnaire, *BFQ*-*C* Big Five Questionnaire for Children

* *p* < .05

** *p* < .01

### Predicting Anxiety from Behavioral Inhibition and Personality Traits

Hierarchical regression analyses were also carried out to examine the unique contributions of sex (step 1), Big Five personality traits (step 2), and behavioral inhibition (step 3) to anxiety disorder symptoms. Predictor variables were found to account for respectively 37 and 45 % of the variance in SCARED-R scores in the non-clinical and clinically anxious group. As shown in Table 4, in both the non-clinical and the clinically anxious group, neuroticism accounted for a unique proportion of the variance in SCARED-R scores (*β’*s being .39 and .26, respectively). As expected, the positive betas indicate that higher levels of neuroticism were associated with higher levels of anxiety symptoms. In the clinically anxious group, conscientiousness was also found to make a unique, negative contribution to anxiety symptoms (*β* = −.37), which means that higher levels of this personality trait were associated with lower levels of anxiety symptoms. Most importantly, behavioral inhibition was also found to explain a substantial, additional proportion of the variance in SCARED-R scores in both the non-clinical and the clinically anxious group (12 and 24 % respectively). The positive betas of respectively .44 and .66 indicate that higher levels of this temperament characteristic were linked to higher levels of anxiety symptoms.

**Table 4:** Main results of the regression analyses predicting children’s DSM-defined anxiety disorder symptoms (SCARED-R) from behavioral inhibition and Big Five personality traits

	B	SE	β	Δ*R*²
*Anxiety disorder symptoms: non*-*clinical children* (*n* = 147)				
Step 1				.01
Sex	2.28	2.02	.08	
Step 2				.24**
BFQ-C extraversion	−.09	.25	−.04	
BFQ-C agreeableness	.17	.23	.07	
BFQ-C conscientiousness	.06	.22	.03	
BFQ-C neuroticism	.88	.18	.39**	
BFQ-C intellect/openness	−.15	.22	−.07	
Step 3				.12**
BIQ behavioral inhibition	.52	.10	.44**	
*Anxiety disorder symptoms: clinically anxious children* (*n* = 45)				
Step 1				.08*
Sex	−2.98	4.87	−.10	
Step 2				.13
BFQ-C extraversion	.18	.52	.06	
BFQ-C agreeableness	.88	.61	.29	
BFQ-C conscientiousness	−.91	.45	−.37*	
BFQ-C neuroticism	.62	.34	.26*	
BFQ-C intellect/openness	.26	.44	.09	
Step 3				.24**
BIQ behavioral inhibition	.72	.19	.66**	

*BIQ* Behavioral Inhibition Questionnaire, *BFQ*-*C* Big Five Questionnaire for Children, *SCARED*-*R* Screen for Child Anxiety Related Emotional Disorders-Revised

* *p* < .10

** *p* < .001

## Discussion

In this study relations between behavioral inhibition, anxiety disorder symptoms and Big Five personality traits were investigated by administering a set of questionnaires in the parents of non-clinical and clinically anxious children. First of all, it was found that parents of clinically anxious children rated their offspring as higher on behavioral inhibition and neuroticism, but lower on extraversion, conscientiousness and intellect/openness as compared to parents of non-clinical youth. The difference between both groups on behavioral inhibition, neuroticism, and extraversion were of course as expected, because there is ample evidence in the literature indicating that children and adolescents with anxiety disorders are characterized by high neuroticism, behavioral inhibition and introversion (the inverse of extraversion) [5, 6, 10].

Further, in keeping with previous studies [9, 10], results indicated that behavioral inhibition in children can best be characterized by low levels of the Big Five personality trait of extraversion (see [24] for similar results in adults). Depue and Collins [25] have suggested that extraversion stems from a biological system promoting active approach and exploration of the environment. As behavioral inhibition is typified by hesitation in exploring the environment, it seems logical that higher scores on this temperamental characteristic are associated with lower scores on the personality trait of extraversion. In addition, studies on the lower-order facets of extraversion have revealed that social inhibition/shyness consistently makes a negative contribution to this supertrait [1].

Besides extraversion, neuroticism was found to make a small but unique contribution to the prediction of behavioral inhibition, although it needs to be mentioned that this was only the case in the sample of non-clinical children. This result corresponds with previous findings indicating that behavioral inhibition at least shares some features with neuroticism [9, 10]. It is unclear why this result was not found in the clinically anxious youth. One explanation is that these children in general already displayed fairly high levels of both neuroticism and behavioral inhibition, as a result of which the positive correlation between these constructs did not emerge in this population.

Although extraversion and neuroticism seem to be important correlates of behavioral inhibition, results from the regression analysis also indicated that these personality traits only accounted for 35–48 % of the variance in behavioral inhibition in respectively non-clinical children and clinically anxious children. This indicates that an inhibited temperament is not fully covered by these basic personality traits, but may also involve a number of other features. An obvious candidate may be the lack of emotion regulation skills, as implicated by a study of Muris and Dietvorst [10], who found that behaviorally inhibited children display low levels of the protective temperament factor of effortful control.

The differences in conscientiousness and intellect/openness between non-clinical and clinically anxious children are more difficult to explain, although it can be argued that children with anxiety problems have more difficulty with fulfilling commitments as a result of their avoidance behavior (which manifest itself in lower conscientiousness scores) and are more reluctant to expose themselves to adventure and new situations (resulting in lower levels of intellect/openness). The regression models further indicated that conscientiousness was positively related to behavioral inhibition in both samples. However, these relations were not found in the correlational analysis. These contrasting findings might be due to shared variance among the personality traits [26].

An additional finding of this investigation was that there were significant differences between boys and girls with regard to some of the Big Five traits. These sex differences were largely in keeping with those observed in previous samples of non-clinical children [9, 17–19], and further demonstrate that differences in personality between males and females already occur at a fairly young age [27], which would also be predicted from theoretical accounts assuming that gender differences have a clear genetic basis [28]. Surprisingly, no sex differences were observed for behavioral inhibition and anxiety symptoms. This is clearly in disagreement with the common observation that girls/women display higher levels of fear and anxiety, and thus are also considered to be more prone to such internalizing symptoms, as compared to boys/men [4]. It is possible that this result occurred because we solely relied on parent data for documenting these phenomena. There is quite some evidence in the literature suggesting that parents are less capable of rating internal symptoms and processes in their offspring [29]. As such the inclusion of self-report data would have yielded important cross-validational or even additional information.

Besides this obvious shortcoming, a number of other limitations of the present study should be noted. First, the fact that this study only relied on data obtained from a single assessor, also implicates that the observed associations were elevated due to shared method variance. In a similar vein, it would have been interesting if we had measured behavioral inhibition by means of an observational procedure in the laboratory. Second, the clinically anxious sample was rather small and consisted of a heterogeneous group of anxiety disorders. It would be worthwhile to explore the links between behavioral inhibition, personality traits and symptoms levels for various types of anxiety disorders, in particular because it has been suggested that an inhibited temperament may be more relevant for social phobia than for other anxiety disorders [30]. Third, a cross-sectional design was used, which does not make it possible to examine the cause-effect relations between behavioral inhibition and neuroticism on the one hand, and children’s anxiety problems on the other.

## Summary

The present results yield further insight in the underlying personality factors of behavioral inhibition, and indicate that extraversion is most strongly associated with an inhibited temperament in both clinically anxious as well as non-clinical children. Further, findings demonstrate that behavioral inhibition explained an additional, unique proportion of the variance in anxiety disorder symptoms of clinically anxious and non-clinical children, even after controlling for personality traits, which implies that behavioral inhibition should be seen as an important correlate of anxiety pathology. This is an important finding, as it suggests that this temperamental characteristic has considerable predictive power for childhood anxiety problems, even beyond common vulnerability factors such as neuroticism and introversion. Behavioral inhibition already becomes manifest at a young age, with some studies indicating that signs of this temperament characteristic are already observable at the age of 4 months [31]. Recent studies have indicated an early intervention program targeting parents are effective in reducing the development of anxiety problems in behaviorally inhibited children [32, 33]. As behavioral inhibition is a vulnerability factor that can be reliably identified with brief and easy-to-administer screening instruments [13, 15, 16], this temperament factor may be an important target for prevention [30].
